# The Effects of Hesperidin on the Healing Process of Cleft Lip Surgical Wounds in Rats: A Histological Evaluation and Therapeutic Analysis

**DOI:** 10.22038/ijorl.2025.85555.3890

**Published:** 2025

**Authors:** Parastoo Namdar, Atena Shiva, Fatemeh Barzegar, Majid Saeedi, Seyyed Mobin Rahimnia, Maziar Khatami, Shahin Arab

**Affiliations:** 1 *Department of Orthodontics, Faculty of Dentistry,* * Dental Research Center,* * Mazandaran University of Medical Sciences, Sari, Iran.*; 2 *Professor of Oral and Maxillofacial Pathology, Faculty of Dentistry, Dental Research Center, Mazandaran University of Medical Sciences, Sari, Iran.*; 3 *Dentist, Mazandaran University of Medical Sciences, Sari, Iran.*; 4 *Professor of Pharmaceutics, * *Mazandaran University of Medical Sciences* *,* * Sari, Iran.*; 5 *Department of Pharmaceutics, Faculty of Pharmacy, Mazandaran University of Medical Sciences, Sari, Iran.*; 6 *Periodontist, Researcher, Mazandaran, Sari, Iran.*; 7 *PhD in Clinical Biochemistry, Faculty of Medicine, Mazandaran University of Medical Sciences, Sari, Iran.*

**Keywords:** Cleft Lip, Hesperidin, Histopathological Techniques, Rats, Wound Healing

## Abstract

**Introduction:**

Cleft lip and palate are the most common congenital craniofacial anomalies, and inadequate treatment of these defects may lead to serious psychosocial and economic consequences. Hesperidin, a flavanone extracted from citrus fruit peels, is a potent antioxidant. However, no study has yet investigated the effects of hesperidin on surgical wound healing in cleft lips. The aim of the present study was to evaluate the histological effects of hesperidin on the healing process of surgically induced cleft lip wounds in rats.

**Materials and Methods:**

In this *in vivo* study, sixteen male Wistar rats were randomly divided into four groups: the control group (normal saline), intervention group 1 (25 mg/kg hesperidin), intervention group 2 (50 mg/kg hesperidin), and intervention group 3 (100 mg/kg hesperidin). A surgical wound was created on the left upper lip of each rat and sutured in two layers. The treatments were administered for 21 days. On day 28 post-surgery, the rats were euthanized, and histopathological analyses were performed to evaluate epithelial proliferation, inflammatory cell density, neovascularization, fibroblast proliferation, and collagen deposition. The samples were stained with hematoxylin and eosin and Masson’s trichrome stains. Statistical significance was set at *P*< 0.05.

**Results:**

The findings showed that the mean scores for fibroblast proliferation, collagen deposition, and inflammatory cell density were significantly higher in the placebo group compared to the 100 mg/kg hesperidin group (*P*= 0.006, *P* =0.009, and *P* = 0.035, respectively). Conversely, epithelial proliferation was significantly higher in the 100 mg/kg hesperidin group compared to the placebo group (*P*= 0.006). However, higher doses of hesperidin resulted in reduced collagen deposition and fibroblast proliferation, although these differences were not statistically significant (*P*> 0.05).

**Conclusion:**

Administration of 100 mg/kg hesperidin decreased fibroblast proliferation, collagen deposition, and inflammatory cell density, while increasing epithelial proliferation during the healing of surgically induced cleft lip wounds in rats. These results suggest that hesperidin may modulate wound repair and contribute to reduced scar formation, which could be particularly beneficial in the aesthetic zone.

## Introduction

Cleft lip and palate are among the most common congenital craniofacial anomalies, occurring in approximately 1 in every 700 live births (1,2). These anomalies, resulting from a failure in the proper fusion of tissues during embryonic development, can present as complete or incomplete defects in the lip and palate (3). Children with cleft lip and palate usually have several problems, such as speech, hearing, feeding, and psychosocial development issues, which may notably affect the quality of life and lead to some social and psychological complications (4,5). 

These patients usually require surgical interventions and multidisciplinary care to restore normal function and aesthetics. Surgical repair of cleft lip, usually performed during the first year of life, aims to reconstruct the lip's muscles and tissues. Nevertheless, one of the more significant post-surgical complications involves the development of hypertrophic scars that can impede functionality and aesthetics (6). 

Hypertrophic scarring results from chronic inflammation, excessive fibroblast proliferation, and the accumulation of extracellular matrix, particularly collagen, at the wound site (7). Current methods for managing hypertrophic scars include corticosteroid injections, laser treatments, and other topical approaches. These methods often have significant side effects and are only moderately effective at reducing scar formation (8). There is, thus, still a need to develop new, safe therapeutic strategies that enhance wound healing while minimizing scar formation (9). Hesperidin is a flavanone of the flavonoid family, which is found mainly in the peels of citrus fruits. Due to its antioxidant, anti-inflammatory, and tissue-regenerative properties, it has been regarded as a promising therapeutic agent for wound healing (10,11). 

By neutralizing free radicals and reducing oxidative stress, hesperidin facilitates angiogenesis and enhances collagen synthesis (12). Previous studies have demonstrated that hesperidin can improve wound healing and reduce scar formation in animal models (13,14). For instance, Gupta et al. (15) demonstrated that a hydrogel containing hesperidin significantly accelerated the healing of full-thickness wounds in animal models. Similarly, Li et al. (11) reported that hesperidin improved the repair of chronic diabetic wounds by modulating molecular pathways associated with angiogenesis and inflammation. Despite these promising findings, no studies have specifically investigated the effects of hesperidin on the healing of cleft lip surgical wounds—a unique and clinically relevant model due to its susceptibility to hypertrophic scarring. Since postoperative scars play a critical role in the psychological and social effects of cleft surgery in childhood Different doses of hesperidin (25, 50, and 100 mg/kg) were used, observing the histological parameters related to the inflammatory cell density, proliferation of fibroblast cells, deposition of collagen fibers, and epithelial proliferation. The findings may support evidence that this compound is useful as an adjunct in improving the quality of wound healing and reducing scar occurrence.This study hypothesizes that topical application of hesperidin, particularly at higher doses, can reduce fibroblast proliferation, collagen deposition, and inflammatory responses while enhancing epithelial regeneration in surgically induced cleft lip wounds in rats.

## Materials and Methods

### Study Design

This experimental and laboratory-based study was conducted at Mazandaran University of Medical Sciences. The sample size calculation was based on the study by Haddadi et al. (16) and the formula below, considering a significance level (α) of 5% and a statistical power (1-β) of 80%. A total of 16 rats, with four rats per group, were selected for the study.

$n=\frac{{\left(z_{1-\frac{\alpha }{2}}+z_{1-\beta }\right)}^{2}*\left(p_{1}q_{1}+p_{2}q_{2}\right)}{{\left(p_{1}-p_{2}\right)}^{2}}$

### Study Population

All experiments were performed on sixteen male Wistar rats aged 6–8 weeks and weighing 320–420 g. The rats were housed under standard laboratory conditions, with the ambient temperature maintained at approximately 22°C, a 12-hour light–dark cycle, and relative humidity controlled within the range of 50–60%. The animals were allowed to acclimatize to the laboratory environment for a minimum of seven days before the initiation of the experiment (17).

### Surgical Procedure

For wound creation, the rats were anesthetized using an intraperitoneal injection of 0.5 mL ketamine hydrochloride (50 mg/kg) and 0.1 mL xylazine hydrochloride (5 mg/kg). After achieving a stable anesthetic plane, the facial fur was shaved. To ensure uniformity, all surgical incisions were made using a sterile triangular metal template (7×7×4 mm), positioned consistently on the left upper lip. The same surgical instruments and technique were used for all animals, and all procedures were performed by a single experienced operator to minimize inter-animal variability (18). The incision sites were sutured in two layers to close the wounds.

### Experimental Groups and Treatments

The rats were randomly assigned to one of four groups. The first group, the control group, had their wounds covered with a normal saline–soaked gauze pad for 5 minutes immediately after closure, serving as the placebo.To prepare 50 g of hesperidin topical gel in three concentrations (25, 50, and 100 mg/g), the polymer Carbopol 941 and triethanolamine were used. Initially, 0.5 g of Carbopol 941 was slowly dissolved in approximately 45 mL of distilled water containing a preservative, preventing lump formation. The mixture was then kept overnight at room temperature to ensure complete polymer hydration. Since hesperidin has low solubility in water, the required amount (depending on the target concentration) was first dissolved in a small volume of ethanol or a water–ethanol mixture and then added to the swollen Carbopol solution. Stirring was continued until a uniform distribution of hesperidin was achieved. Subsequently, triethanolamine was added dropwise to adjust the pH to approximately 6.8–7.0, resulting in the formation of a clear, viscous gel. If necessary, the final volume was adjusted to 50 mL with distilled water, and the mixture was gently stirred to eliminate air bubbles and ensure homogeneity. 

The gels were then transferred into aluminum tubes and stored at room temperature until use.

The second group, intervention group 1 (Hesperidin 25), received topical application of 25 mg/kg hesperidin gel for 21 days following surgery. The third group, intervention group 2 (Hesperidin 50), was treated with 50 mg/kg hesperidin gel, applied topically for 21 consecutive days after surgery. The fourth group, intervention group 3 (Hesperidin 100), received the highest concentration of hesperidin gel (100 mg/kg) topically for the same duration. All treatments were administered once daily throughout the study period.

### Histological Assessment

On day 28 post-surgery, all rats were euthanized humanely using CO₂ asphyxiation. The surgical wounds were excised and fixed in 10% neutral-buffered formalin for 24 hours. The tissue specimens were then processed for routine histological evaluation, including fixation, dehydration, embedding, and sectioning. Sections of 5 μm thickness were prepared and stained with hematoxylin and eosin (H&E) for general histological evaluation and with Masson’s trichrome stain to assess collagen deposition.

A blinded pathologist evaluated the histological parameters of wound healing, including:

- Epithelial proliferation
- Inflammatory cell density
- Neovascularization (new capillary formation)
- Fibroblast proliferation
- Collagen deposition

Each parameter was scored on a scale from 0 to 3 (none, mild, moderate, or marked). The average scores were calculated for statistical analysis. Additionally, the presence or absence of necrosis and abscess formation was recorded. Table 1 summarizes the key variables and the methods used for their assessment (19).

**Table 1:** Summary of key variables and assessment methods

**Variable**	**Assessment Method**	**Scale**	**Staining Technique**
Inflammatory Cell Density	Blinded histological scoring	Ordinal (0–3)	Hematoxylin-Eosin
New Capillary Formation	Blinded histological scoring	Ordinal (0–3)	Hematoxylin-Eosin
Epithelial Proliferation	Blinded histological scoring	Ordinal (0–3)	Hematoxylin-Eosin
Fibroblast Proliferation	Blinded histological scoring	Ordinal (0–3)	Hematoxylin-Eosin
Collagen Deposition	Blinded histological scoring	Ordinal (0–3)	Masson's Trichrome

### Statistical analysis

Data were analyzed utilizing SPSS software (version 25). Descriptive statistics, encompassing frequency, percentage, mean, standard deviation, median, and range, were employed to summarize the dataset. For the assessment of differences between groups regarding continuous variables, the Kruskal-Wallis test was applied, followed by Dunn’s post-hoc test with Bonferroni correction to control for multiple comparisons. A p-value of < 0.05 was considered statistically significant for all tests.

## Results

This study analyzed 16 samples from four groups regarding fibroblast proliferation, collagen deposition, inflammatory cells, and epithelial cell proliferation. All these variables were ordinal. The results are as follows:

*Comparison of fibroblast proliferation scores among groups*

Table 2 summarizes the distribution of fibroblast proliferation scores across four experimental groups: Placebo, Hesperidin 25, Hesperidin 50, and Hesperidin 100. 

Fibroblast proliferation was assessed in three categories: mild (+1), moderate (+2), and marked (+3). The results reveal distinct patterns of proliferation associated with each treatment.

**Table 2:** Comparison of fibroblast proliferation scores among groups

**Group**	**Mild (+1)**	**Moderate (+2)**	**Marked (+3)**	**Total**	**Mean Scores**	**Mean Ranks**
Placebo	0 (0%)	0 (0%)	4 (100%)	4	3.00	13.5
Hesperidin 25	0 (0%)	2 (50%)	2 (50%)	4	2.50	10.25
Hesperidin 50	0 (0%)	4 (100%)	0 (0%)	4	2.00	7.00
Hesperidin 100	3 (75%)	1 (25%)	0 (0%)	4	1.25	3.25
Total	3 (18.8%)	7 (43.8%)	6 (37.5%)	16	2.19	-
Kruskal-Wallis Test	χ² = 11.87 P = 0.008					

In the placebo group, all four samples (100%) exhibited marked (+3) fibroblast proliferation, reflecting the highest level of activity among all groups. This group also had the highest mean score (3.00) and mean rank (13.5). In the Hesperidin 25 group, the scores were evenly distributed, with 50% of samples classified as moderate (+2) and 50% as marked (+3). This group demonstrated intermediate proliferation levels, with a mean score of 2.50 and a mean rank of 10.25.

The Hesperidin 50 group showed consistent moderate (+2) proliferation across all samples (100%), resulting in a mean score of 2.00 and a mean rank of 7.00. In contrast, the Hesperidin 100 group exhibited the lowest fibroblast proliferation, with 75% of samples categorized as mild (+1) and 25% as moderate (+2). This group had the lowest mean score (1.25) and mean rank (3.25).

Across all 16 samples, 18.8% were categorized as mild (+1), 43.8% as moderate (+2), and 37.5% as marked (+3). The overall trend suggests that increasing doses of hesperidin are associated with lower fibroblast proliferation scores. Statistical analysis using the Kruskal–Wallis test revealed a significant difference in proliferation scores among the groups (χ² = 11.87, P = 0.008), indicating that these variations were not due to random chance.

These findings suggest a dose-dependent effect of hesperidin on fibroblast proliferation. The placebo group, which received no hesperidin, showed the highest proliferation, whereas the Hesperidin 100 group, which received the highest dose, showed the lowest proliferation. This highlights the potential suppressive effect of hesperidin on fibroblast activity, particularly at higher doses.

Dunn’s post hoc test was performed to compare fibroblast proliferation scores among the placebo and the three hesperidin-treated groups. 

The results indicated no significant differences between the placebo and the Hesperidin 25 or Hesperidin 50 groups (P = 0.99 and P = 0.224, respectively). However, a significant difference was observed between the placebo and Hesperidin 100 groups (P = 0.006*), suggesting that the highest dosage of hesperidin significantly decreased fibroblast proliferation.

In comparisons among the hesperidin-treated groups, no significant differences were found between Hesperidin 25 and Hesperidin 50 (P = 0.99), between Hesperidin 25 and Hesperidin 100 (P = 0.150), or between Hesperidin 50 and Hesperidin 100 (P = 0.99). Overall, these findings indicate that while the highest dose of hesperidin exerted a significant effect on fibroblast proliferation, lower doses did not significantly reduce cellular activity.

This context is essential for evaluating the therapeutic potential of hesperidin and its dose-dependent suppressive effects on cellular behavior. It can be observed that in the placebo group, 100% of proliferation scores were categorized as marked, indicating the highest response. The Hesperidin 25 group displayed a distribution across all categories—marked, moderate, and mild. In the Hesperidin 50 group, proliferation predominantly fell into the moderate category, with no scores in the mild or marked categories. The Hesperidin 100 group exhibited scores only in the moderate and mild categories, with no marked scores, suggesting a reduction in proliferation at this highest dose.

Overall, as the dosage of hesperidin increased from 25 to 100 mg/kg, there was a clear trend of reduced fibroblast proliferation, shifting from marked to moderate and finally to mild. The placebo group demonstrated the highest proliferation, while the Hesperidin 100 group showed the lowest response. These findings suggest that hesperidin may exert a dose-dependent inhibitory effect on fibroblast proliferation, warranting further analysis to confirm statistical significance and explore potential mechanisms.

*Comparison of Collagen Deposition Scores Among Groups*

Table 3 summarizes the distribution of collagen deposition scores across the four experimental groups: Placebo, Hesperidin 25, Hesperidin 50, and Hesperidin 100. Collagen deposition was evaluated in three categories: mild (+1), moderate (+2), and marked (+3). The results reveal distinct patterns of collagen deposition associated with each treatment.

**Table 3:** Comparison of collagen deposition scores among groups

**Group**	**Mild (+1)**	**Moderate (+2)**	**Marked (+3)**	**Total**	**Mean Scores**	**Mean Ranks**
Placebo	0 (0%)	0 (0%)	4 (100%)	4	3.00	13.00
Hesperidin 25	0 (0%)	2 (50%)	2 (50%)	4	2.50	9.75
Hesperidin 50	0 (0%)	3 (75%)	1 (25%)	4	2.25	8.13
Hesperidin 100	3 (75%)	1 (25%)	0 (0%)	4	1.25	3.13
Total	3 (18.8%)	6 (37.5%)	7 (43.8%)	16	2.25	-
Kruskal-Wallis Test	χ² = 10.43 P = 0.015					

In the Placebo group, all four samples (100%) exhibited marked (+3) collagen deposition, representing the highest level among the groups. This group had the highest mean score (3.00) and mean rank (13.00). In the Hesperidin 25 group, collagen deposition scores were evenly distributed, with 50% of samples classified as moderate (+2) and 50% as marked (+3). This group exhibited intermediate levels of collagen deposition, with a mean score of 2.50 and a mean rank of 9.75. The Hesperidin 50 group showed 75% of samples classified as moderate (+2) and 25% as marked (+3), resulting in a mean score of 2.25 and a mean rank of 8.13.

The Hesperidin 100 group demonstrated the lowest levels of collagen deposition, with 75% of samples categorized as mild (+1) and 25% as moderate (+2). This group had the lowest mean score (1.25) and mean rank (3.13). Across all 16 samples, 18.8% were classified as mild (+1), 37.5% as moderate (+2), and 43.8% as marked (+3). The Kruskal–Wallis test revealed a statistically significant difference in collagen deposition scores among the groups (χ² = 10.43, P = 0.015), indicating that these variations were not due to chance. These findings suggest a dose-dependent effect of hesperidin on collagen deposition, with higher doses leading to reduced deposition. The placebo group, which received no hesperidin, exhibited the highest collagen deposition, while the Hesperidin 100 group, which received the highest dose, showed the lowest deposition, emphasizing the potential suppressive effect of hesperidin on collagen synthesis. Dunn’s post-hoc test was used to analyze collagen deposition scores across the treatment groups, including the placebo and the three hesperidin dosage groups. 

The following findings provide insights into the efficacy of hesperidin in reducing fibroblast proliferation.

The analysis of collagen deposition revealed significant findings regarding the effects of hesperidin dosage. Comparisons between the placebo and Hesperidin 25 and Hesperidin 50 groups showed no statistically significant differences (P = 0.99 and P = 0.711, respectively). However, a significant difference was observed between the placebo and Hesperidin 100 groups (P = 0.009), indicating that the highest concentration of hesperidin significantly influences collagen deposition. In inter-group comparisons among the hesperidin dosages, no significant differences were found. Specifically, Hesperidin 25 did not differ significantly from Hesperidin 50 (P = 0.99) or Hesperidin 100 (P = 0.203), and there was no significant difference between Hesperidin 50 and Hesperidin 100 (P = 0.656). Overall, the key insight is that while lower dosages (25 and 50 mg/kg) did not show significant effects compared to the placebo or to each other, the 100 mg/kg dosage demonstrated a pronounced impact in reducing collagen deposition, suggesting a potential dose-dependent effect. The observations indicate that the placebo 

group had the highest collagen deposition, with 100% of scores in the marked category. As the hesperidin dosage increased, scores progressively shifted from marked to moderate and mild, particularly in the Hesperidin 100 group, which showed no marked scores. This suggests a dose-dependent inhibitory effect of hesperidin on collagen deposition, paralleling the trends observed in fibroblast proliferation. Further analysis is warranted to confirm these findings and explore their potential clinical implications.

*Comparison of inflammatory cell scores among groups*

Table 4 presents the distribution of inflammatory cell scores across four experimental groups: Placebo, Hesperidin 25, Hesperidin 50, and Hesperidin 100. Inflammatory cell scores were classified into three categories: mild (+1), moderate (+2), and marked (+3). The data reveal distinct patterns of inflammatory cell presence associated with each treatment.

**Table 4:** Distribution of inflammatory cell scores among groups

**Group**	**Mild (+1)**	**Moderate (+2)**	**Marked (+3)**	**Total**	**Mean Scores**	**Mean Ranks**
Placebo	0 (0%)	4 (100%)	0 (0%)	4	2.00	13
Hesperidin 25	3 (75%)	1 (25%)	0 (0%)	4	1.25	7
Hesperidin 50	2 (50%)	2 (50%)	0 (0%)	4	1.50	9
Hesperidin 100	4 (100%)	0 (0%)	0 (0%)	4	1.00	5
Total	9 (56.3%)	7 (43.8%)	0 (0%)	16	1.44	-
Kruskal-Wallis Test	χ² = 8.33 P = 0.040					

In the Placebo group, all samples (100%) were classified as moderate (+2), indicating the highest inflammatory response among the groups. This group had a mean score of 2.00 and the highest mean rank (13.00). The Hesperidin 25 group showed a significant reduction in inflammation, with 75% of samples categorized as mild (+1) and 25% as moderate (+2). This group had a mean score of 1.25 and a mean rank of 7.00. Similarly, in the Hesperidin 50 group, 50% of the samples were mild (+1), and 50% were moderate (+2), resulting in a mean score of 1.50 and a mean rank of 9.00.

The Hesperidin 100 group exhibited the lowest inflammatory response, with all samples (100%) categorized as mild (+1). This group had the lowest mean score (1.00) and the lowest mean rank (5.00). Overall, across all 16 samples, 56.3% were categorized as mild (+1) and 43.8% as Moderate (+2), with no samples falling into the marked (+3) category. Statistical analysis using the Kruskal-Wallis test revealed a significant difference in inflammatory cell scores among the groups (χ²=8.33, P=0.040). These findings suggest that higher doses of Hesperidin are associated with reduced inflammatory cell presence. 

The Placebo group, which received no Hesperidin, showed the highest inflammatory response, whereas the Hesperidin 100 group, receiving the highest dose, demonstrated the lowest inflammatory response. This highlights the potential anti-inflammatory effect of Hesperidin, particularly at higher doses. This study examines the differential impacts of varying hesperidin concentrations (25, 50, and 100 units) on inflammatory cell scores compared to a placebo group. *The visualization of the inflammatory cell scores highlights a statistically significant difference between the placebo group and the 100-unit hesperidin group (P=0.035). *

*This indicates that the highest hesperidin concentration might have an anti-inflammatory effect. Comparisons between other groups showed no significant differences, suggesting that the 100-unit dosage uniquely impacts inflammatory cell responses.*

*Comparison of epithelial proliferation scores among groups*

Table 5 presents the distribution of epithelial proliferation scores across four experimental groups: Placebo, Hesperidin 25, Hesperidin 50, and Hesperidin 100. Epithelial proliferation was categorized into three levels: mild (+1), moderate (+2), and marked (+3). The table highlights distinct patterns of epithelial proliferation associated with the different treatment groups.

**Table 5:** Distribution of epithelial proliferation scores among groups

**Group**	**Mild (+1)**	**Moderate (+2)**	**Marked (+3)**	**Total**	**Mean Scores**	**Mean Ranks**
Placebo	4 (100%)	0 (0%)	0 (0%)	4	1.00	3.5
Hesperidin 25	2 (50%)	2 (50%)	0 (0%)	4	1.50	6.75
Hesperidin 50	0 (0%)	4 (100%)	0 (0%)	4	2.00	10
Hesperidin 100	0 (0%)	1 (25%)	3 (75%)	4	2.75	13.75
Total	6 (37.5%)	7 (43.8%)	3 (18.8%)	16	1.81	-
Kruskal-Wallis Test	χ² = 11.87	P = 0.008				

In the Placebo group, all samples (100%) were categorized as mild (+1), representing the lowest level of epithelial proliferation among the groups. This group had the lowest mean score (1.00) and mean rank (3.5). In the Hesperidin 25 group, 50% of the samples were classified as mild (+1) and 50% as moderate (+2), resulting in a mean score of 1.50 and a mean rank of 6.75. The Hesperidin 50 group showed an increase in proliferation, with all samples (100%) classified as moderate (+2). This group had a mean score of 2.00 and a mean rank of 10.00.

The Hesperidin 100 group exhibited the highest epithelial proliferation levels, with 75% of the samples categorized as marked (+3) and 25% as moderate (+2). This group had the highest mean score (2.75) and mean rank (13.75). Overall, across all 16 samples, 37.5% were classified as mild (+1), 43.8% as moderate (+2), and 18.8% as marked (+3). Statistical analysis using the Kruskal-Wallis test revealed a significant difference in epithelial proliferation scores among the groups (χ²=11.87, P=0.008). These findings suggest that Hesperidin treatment is associated with increased epithelial proliferation in a dose-dependent manner. While the Placebo group showed the lowest proliferation, the Hesperidin 100 group, receiving the highest dose, demonstrated the highest levels of epithelial proliferation. This highlights a stimulatory effect of Hesperidin on epithelial cell activity, particularly at higher doses. This study examines the differential impacts of varying hesperidin concentrations (25, 50, and 100 units) on Epithelial Proliferation Scores compared to a placebo group.

The analysis revealed no statistically significant differences between the placebo and lower doses of Hesperidin (25 and 50 units). However, a significant effect was observed with the highest dose (100 units) compared to the placebo (P=0.006) and indicating that only the 100-unit dose had a pronounced impact on epithelial proliferation, It shows a dose-dependent effect of potential epithelial regeneration enhancement at higher concentrations.

**Fig. 1:**
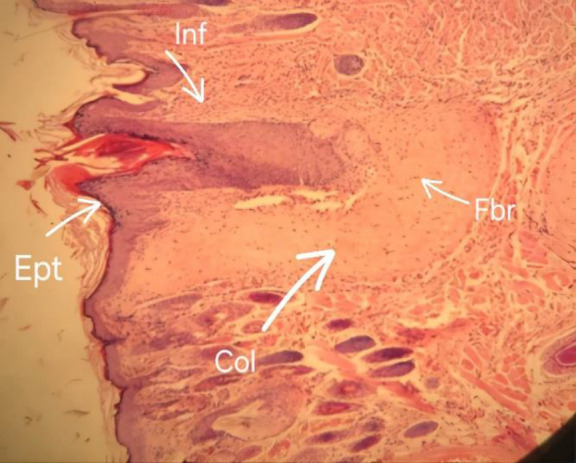
Histopathological analysis of Epithelial proliferation (Ept), inflammatory cell distribution (Inf), collagen deposition (col), fibroblast proliferation (Fbr) in the tissue sections of Hesperidin 25 on the 28th postoperative day H&E staining with 100x magnification

**Fig. 2:**
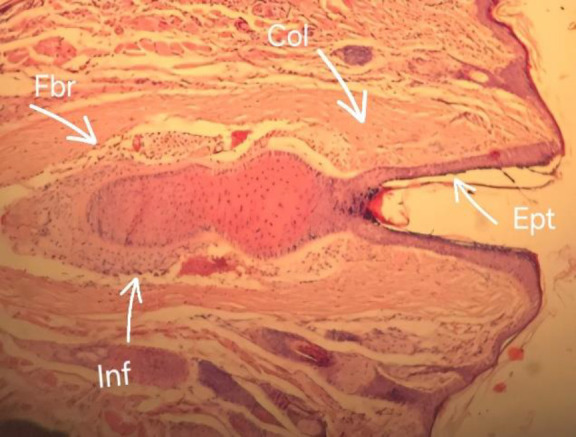
Histopathological analysis of Epithelial proliferation (Ept), inflammatory cell distribution (Inf), collagen deposition (col), fibroblast proliferation (Fbr) in the tissue sections of Hesperidin 50 on the 28th postoperative day H&E staining with 100x magnification

**Fig 3:**
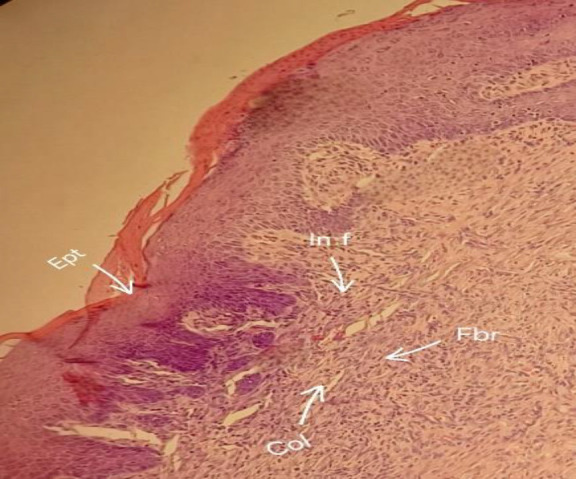
Histopathological analysis of Epithelial proliferation (Ept), inflammatory cell distribution (Inf), collagen deposition(col), fibroblast proliferation (Fbr) in the tissue sections of Hesperidin 100 on the 28th postoperative day H&E staining with 100x magnification

 As the Hesperidin dose increased, epithelial proliferation scores shifted from mild and moderate to marked (high proliferation). The Placebo group showed the lowest proliferation with all scores in the miled category. In Hesperidin 25, most scores remained mild to moderate, while in Hesperidin 50, the scores were moderate. In Hesperidin 100, most scores shifted to maked.  Histopathological analysis of Epithelial proliferation (Ept), inflammatory cell distribution (Inf), collagen deposition (col), fibroblast proliferation (Fbr) in the tissue sections of Hesperidin (Fig.1), 50(Fig. 2) and Hesperidin 100 (Fig.3) on the 28th postoperative day heamatoxylin and eosin staining with 100x magnification is indicated.

(Figures 1-3) demonstrates the structural changes in epithelial tissue proliferation across experimental groups, emphasizing the proliferative effects of hesperidin at varying doses. demonstrates the structural changes in epithelial tissue proliferation across experimental groups, emphasizing the proliferative effects of hesperidin at varying doses. This image highlights epithelial proliferation with varying thickness and structural changes. Noticeable is the hyperplastic appearance of the epithelial layer, suggesting active proliferation. This structural alteration corresponds with moderate-to-marked proliferation levels observed in the experimental groups, especially in the high-dose hesperidin group.

## Discussion

The current study aimed to histologically evaluate the effect of hesperidin at different doses (25, 50, and 100 mg/kg) on the healing of surgical cleft lip wounds in rats. The findings revealed that the mean scores for fibroblast proliferation, collagen deposition, and inflammatory cells were significantly higher in the saline-treated (placebo) group compared to the group treated with 100 mg/kg hesperidin. However, the mean epithelial proliferation score was significantly higher in the 100 mg/kg hesperidin group than in the placebo group. The parameters assessed showed no significant differences among the other groups.

Excessive fibroblast proliferation and collagen deposition lead to the formation of hypertrophic scars. Therefore, increased fibroblast proliferation and collagen deposition indicate greater scar formation (7). In contrast to the present study, research by Duman et al. reported that topical application of 2.5% folinic acid in mice increased fibroblast proliferation and collagen deposition, accelerating wound healing but also raising the risk of hypertrophic scar formation (20). Conversely, a study by Jin et al. demonstrated that kaempferol inhibits fibroblast collagen synthesis, proliferation, and activation by selectively binding to TGFβRI, suppressing TGF-β/Smad signaling, and consequently reducing hypertrophic scar formation in a murine model (21). These findings align with the current study.

Reconstruction of the lip and continuity of the orbicularis oris muscle are critical in the treatment of patients with cleft lips. However, scar formation remains a common complication of this surgery. Achieving aesthetic and functional outcomes while minimizing scar formation is a primary goal of such procedures. Various agents have been investigated to enhance wound healing and reduce hypertrophic scar formation (22). Recent studies have reported that intralesional injection of botulinum toxin type A effectively inhibits hypertrophic scars (8,23); however, this treatment does not lead to the effective regeneration of healthy skin tissue (24).

Scar formation is a significant component of mammalian tissue repair; nevertheless, impaired resolution can lead to excessive extracellular matrix (ECM) accumulation, resulting in pathological scarring. Tissue damage repair aims to restore tissue integrity through complex, tightly regulated biological processes involving collaboration among multiple cell types, growth factors, cytokines, and ECM components. Scarless wound healing is essential for both functional and aesthetic outcomes (25). Hypertrophic scars typically develop within one to three months post-injury. Numerous factors, including race, age, genetics, hormone levels, atopy, and individual immune responses, contribute to hypertrophic scar formation. Additionally, the type of injury, wound size and depth, anatomical location, and mechanical tension on the wound play critical roles. Other factors, such as bacterial colonization and wound infection, also contribute to hypertrophic scar development (26). When the skin is injured, the initial inflammatory cascade is activated, recruiting various inflammatory cells to the wound site, where they release cytokines (27). These cytokines stimulate keratinocyte and fibroblast migration to the wound site, followed by their proliferation, which begins 4–5 days later. Fibroblasts secrete ECM proteins such as fibronectin, collagen, and hyaluronic acid, forming granulation tissue (28). Hypertrophic scars exhibit higher vascular density than normal scars. About a week after wound stabilization, some fibroblasts differentiate into myofibroblasts, which also secrete ECM proteins, including collagen types I and III. Myofibroblasts play a key role in wound edge contraction and wound size reduction. Concurrently, keratinocyte proliferation at the wound margins initiates re-epithelialization (29). As re-epithelialization progresses, blood vessel numbers decline, leading to fibroblast and myofibroblast apoptosis and cessation of wound contraction. Consequently, mature scar tissue contains few fibroblasts (30). However, hypertrophic scar formation results from an imbalance between ECM synthesis and degradation during wound healing. Excess inflammatory cytokines, including interleukin (IL)-1β, IL-6, and tumor necrosis factor-alpha, not only enhance fibroblast proliferation and ECM synthesis but also inhibit collagenase activity and increase collagenase inhibitor production. These events result in abnormal collagen composition and eventually lead to scar formation. Complete wound healing time is widely recognized as the most critical predictor of hypertrophic scar development (24). According to the current findings, the use of hesperidin at 100 mg/kg significantly reduced fibroblast proliferation, collagen deposition, and inflammatory cells in wounds, thereby reducing hypertrophic scar formation.While Yang et al. (31) demonstrated the scar-reducing potential of hesperidin in a rabbit ear model, the current study uniquely confirms its therapeutic benefit in surgically induced cleft lip wounds in rats. Unlike earlier models focusing on generic dermal wounds, this model closely mimics the clinical setting of cleft repair, making the results more translational.

In this study, hesperidin at doses of 25 and 50 mg/kg did not significantly reduce scar formation compared to the control group. However, it is noteworthy that collagen deposition and fibroblast proliferation decreased with increasing doses of hesperidin, although these differences were not statistically significant. Previous studies have shown that hesperidin prevents cancer cell proliferation, induces apoptosis, and inhibits angiogenesis (32,33). Additionally, it can inhibit ECM component synthesis or secretion induced by TGF-β1 in human fetal lung fibroblasts (34). Several studies have demonstrated that hesperidin exhibits protective effects against chemically induced liver cancer, potentially by inhibiting the TGF-β1/Smad3 signaling pathway (35,36). The TGF-β/Smad signaling pathway is recognized as a crucial factor in hypertrophic scarring, and hesperidin effectively inhibits this pathway. Thus, it can be hypothesized that hesperidin has the potential to prevent scar formation (37,38).

## Conclusion

This study investigated the effects of hesperidin at various doses on the healing of cleft lip surgical wounds in a rat model, focusing on its potential to reduce hypertrophic scar formation. The results demonstrated that administration of 100 mg/kg hesperidin significantly reduced fibroblast proliferation, collagen deposition, and inflammatory cell infiltration compared to the control group, indicating a marked decrease in scar formation. Moreover, epithelial proliferation was significantly enhanced in the 100 mg/kg hesperidin group, highlighting improved epithelial regeneration. While incremental increases in hesperidin dosage were associated with reductions in fibroblast proliferation, collagen deposition, and inflammatory cell infiltration, these changes did not reach statistical significance at doses below 100 mg/kg. These findings suggest that hesperidin, particularly at a dose of 100 mg/kg, is effective in enhancing the wound healing process and minimizing hypertrophic scar formation following cleft lip surgery.

The limited availability of prior research on hesperidin’s effects on wound healing posed challenges in contextualizing the findings within existing literature. Furthermore, the study did not evaluate the effects of hesperidin over a prolonged period, leaving uncertainties regarding its long-term efficacy and safety in scar management.

This study is limited by its relatively small sample size (n=16), which may impact statistical power and generalizability. The lack of long-term follow-up also limits conclusions regarding the durability of histological improvements. Molecular pathway analyses were not performed and should be included in future studies.

To address these limitations, future research should incorporate longitudinal designs to assess the histopathological and clinical effects of hesperidin throughout the wound healing process. Expanding the sample size will enhance the reliability and reproducibility of the findings, while clinical trials will be essential for determining the translational applicability of hesperidin in human populations. Additionally, comprehensive investigations of the underlying molecular mechanisms-such as hesperidin’s role in modulating TGF-β/Smad signaling and its interaction with extracellular matrix remodeling-could further elucidate its therapeutic potential.

In summary, hesperidin shows promise as an effective agent for improving wound healing outcomes and reducing hypertrophic scar formation. These findings strongly support the therapeutic potential of hesperidin, particularly at higher doses, in enhancing epithelial regeneration and minimizing fibrotic responses following cleft lip surgery. Future studies should focus on clinical trials in human patients and mechanistic investigations evaluating hesperidin’s interaction with key signaling pathways.

### Ethical Considerations

 The study protocol was approved by the Ethics Committee of Mazandaran University of Medical Sciences (Ethics Code: IR.MAZUMS.REC.1402.19001).

### Conflicts of Interest

 The authors declare that there is no conflict of interest.
